# Ethnopharmacological Knowledge and Antioxidant Propensities of *Argania spinosa* L. from Morocco

**DOI:** 10.1155/2024/6795451

**Published:** 2024-07-29

**Authors:** Mohamed El Yamani, Rachid Ijjouk, Kholoud Kahime, Yahia Rharrabti

**Affiliations:** ^1^ Laboratory of Applied Sciences for the Environment and Sustainable Development Essaouira School of Technology Cadi Ayyad University, Km 9, Route d'Agadir, BP. 383, Essaouira Al Jadida, Morocco; ^2^ Laboratory of Natural Resources and Environment Polydisciplinary Faculty of Taza Sidi Mohamed Ben Abdellah University, Taza-Gare, BP. 1223, Taza, Morocco

## Abstract

This work aims to merge ethnopharmacological knowledge with biochemical analysis to enrich our understanding of the significance of the argan tree (*Argania spinosa* (L.) Skeels) and to valorize its crucial role in the province of Essaouira (Morocco). First, a survey was conducted using semistructured interviews with 325 informants from Essaouira province between February and April 2023. The interviews covered sociodemographic data and information on argan tree uses, whether for therapeutic, cosmetic, or food purposes (i.e., applications, parts used, preparation, and administration). Second, phenolic extracts were prepared from various parts of the argan tree (i.e., leaves, kernels, nut shells, press cake, and oil) and then assessed for their antioxidant potential to scientifically validate their traditional uses. The evaluation of antioxidant activity focused on their free radical scavenging and reducing capacities, using DPPH and FRAP assays. Findings confirmed the cultural significance of the argan tree for the local population, as well as their strong dependence on its products. Indeed, it was noted that argan-based products are widely favored in traditional cuisine, with a prevalence of 83.4%; Amlou is the most commonly consumed food. Therapeutic and cosmetic applications accounted for 48.6% and 28.0%, respectively, predominantly for treating skin and subcutaneous issues (69.5%) and diabetes (19.7%). Argan oil was the most cited argan product used, often consumed raw (97.5%), followed by almonds (22.8%). Cataplasm (26.1%) and maceration (24.6%) were preferred for argan derivative preparation. External application (50.1%) was the primary administration method, followed by oral consumption (38.1%) and massage (27.7%). For *in vitro* assays, the argan tree could prove to be a promising source of phenolic compounds, especially in the leaves (>4 times richer than other parts, 231.046 ± 5.090 mg GAE/g DW). DPPH and FRAP tests demonstrated notable antiradical potential and reducing power, concentration-dependent. Leaf-derived phenolic extracts exhibited the highest free radical scavenging potential (IC50 = 0.589 ± 0.005 mg/ml) and the best reducing capacity (IC50 = 0.420 ± 0.005 mg/ml), although these potencies remained below the standard used. This study represents valuable documentation that can serve to preserve information on the use of argan products while exploring their phytochemical and pharmacological properties.

## 1. Introduction

The argan tree (*Argania spinosa* (L.) Skeels), the only representative of the *Sapotaceae* family, is a crucial endemic and medicinal species of significant ecological, socioeconomic, and cultural relevance in southwestern Morocco [1, 2]. This tree is renowned for its resilience in severe environments and for its role in sustaining local communities [3]. It covers an area of 830,000 hectares, starting from Safi in the north and moving south to the edge of the Sahara, with the main zone lying southeast of Essaouira on the Souss plain. The argan ecological range has extended well beyond its primary native habitat in southwest Morocco, covering relict sites in the northeast and west (Beni-Znassen, Oued-Cherrat, and the Bouareg plain) [4–6]. This noteworthy adaptive capacity emphasizes its resistance and resilience, thereby ensuring its ability to thrive in diverse environments and cope with changing climatic conditions [7]. For Essaouira province, the argan tree occupies 136,750 hectares, accounting for 20% of the national argan grove acreage and 7% of the national forest area. The Haha tribe holds 84% of this area, while the remaining 16% belongs to the Chiadma tribe [3, 8]. The argan orchards in the province of Essaouira generate an annual average of 450 kg of fresh fruit per hectare, resulting in an oil output of approximately 1,138 tons. This production meets 36% of the global needs of the provincial population [8]. Besides its recognized role in maintaining ecological equilibrium and its economic value in providing a crucial source of income and sustenance for the local population, the argan has long been revered for its extensive contributions to various facets of human life, covering health, medicine, food, and cosmetics [9, 10]. Its importance is particularly noteworthy in the province of Essaouira (Morocco), making it a subject of great interest for ethnopharmacological and biochemistry studies.

During the last few years, increasing interest has been focused on the study of traditional medicinal plants and their ethnopharmacological knowledge, both for their potential therapeutic applications and their cultural significance [11–13]. Notably, *Argania spinosa* (L.) Skeels is considered particularly important in the Essaouira province (Morocco). Indeed, it represents an integral component of the local ecosystem, as well as of the cultural heritage and traditional practices associated with the province [14]. Local communities have accumulated rich knowledge and expertise in using various argan tree parts for medicinal, cosmetic, and dietary purposes. Argan oil, prized for its high nutritional value, is used in food [15], cosmetics [16, 17], and therapeutic purposes [18–20]. Additionally, the various parts of the tree (i.e., leaf, seed, kernel, pulp, bark, wood, and root) are also used to treat wide-ranging disorders, including diabetes, rheumatism, eczema, dry skin, burns, hypercholesterolemia, gastritis, ulcers, dysentery, headaches, and fever, as well as for purposes related to hydration, regeneration, antiaging, hair, and face care [18, 21–25]. Traditional knowledge of the beneficial properties of *Argania spinosa* (L.) Skeels has been inherited through generations among the indigenous population of Essaouira [26, 27]. These communities have also extensive expertise in the uses, preparation methods, and use doses for the various parts of the plant. Documenting and analyzing this traditional knowledge, also known as ethnopharmacology, could offer great opportunities in terms of preserving cultural heritage and discovering new bioactive compounds having therapeutic potential. Nevertheless, for all the cultural and medicinal relevance of *Argania spinosa* (L.) Skeels in the province of Essaouira, ethnopharmacological investigations are still relatively limited.

Each part of the argan tree, from the fruit pulp to the precious oil extracted from the almonds, as well as the leaves and roots, holds valuable properties that promote human health and well-being. Thanks to their wealth of essential nutrients such as vital fatty acids, antioxidants, vitamins, and minerals, these components of the argan tree exert antioxidant, antibacterial, antifungal, anti-inflammatory, antidiabetic, anticancer, hydrating, and regenerative actions [10, 28–32]. World-renowned for their exceptional properties, argan trees have increasingly been explored for scientific and commercial purposes. Indeed, among the most appreciated attributes of the argan tree is its impressive antioxidant potential, which remains of great relevance in terms of its therapeutic and protective effects. Antioxidants are involved in neutralizing harmful free radicals, thereby reducing oxidative stress and exposure to chronic diseases notably cardiovascular disorders, cancer, and neurodegenerative disorders [33–35].

Several studies have investigated the phytochemical composition and antioxidant capacity of various parts of the argan tree and found a variety of bioactive metabolites, such as phenolic compounds, tocopherols, carotenoids, and saponins [36, 37]. Argan fruits, rich in antioxidants, contain substances that protect cells from damage, prevent inflammation, and improve skin health [10, 38]. High levels of polyphenols, flavonoids, and tannins in argan leaves have been shown to confer antioxidant abilities and may be beneficial in dealing with oxidative stress [32, 37, 39, 40]. Argan nuts and oil naturally contain many valuable antioxidants, including vitamin E, sterols, and phenolic compounds, which help protect the body from harmful substances [28, 31, 41–43]. These antioxidant properties have made argan oil an exceptionally beneficial product. Additionally, bark, branches, and flower buds of the argan tree have been also studied for their antioxidant properties and found to contain substances such as phenolic compounds and flavonoids able to protect cells against oxidative damage and oxidation-related diseases [36, 44, 45]. It is therefore absolutely crucial to investigate the antioxidant capacities of the various argan tree components. In turn, this will help both to preserve this invaluable natural asset and to unlock the potential for future research and development in the cosmeceutical, pharmaceutical, and nutraceutical fields. Ultimately, this will support sustainable development, health, and well-being.

Although argan (*Argania spinosa* (L.) Skeels) is widely used and recognized as a valuable medicinal resource, there are few studies to support traditional claims about its pharmacological properties and to explore its chemical composition and properties, as well as its biological activities. However, such studies offer a promising pathway to fill these gaps and discover the potential applications of the argan in modern medicine. Within the above context, this study aims to combine ethnopharmacological knowledge and biochemical exploration to better understand *Argania spinosa* (L.) Skeels from the province of Essaouira (Morocco). First, we focused on capturing and documenting the traditional uses, preparation methods, cultural significance, and valuable heritage associated with the use of the argan tree in food, cosmetics, and traditional medicine. Second, we attempted to provide scientific validation for its traditional uses by investing in the antioxidant potential (antifree radical and antioxidant capacities) of phenolic extracts from argan products and derivatives.

## 2. Materials and Methods

### 2.1. Ethnopharmacological Survey

#### 2.1.1. Study Area

**Figure 1:**
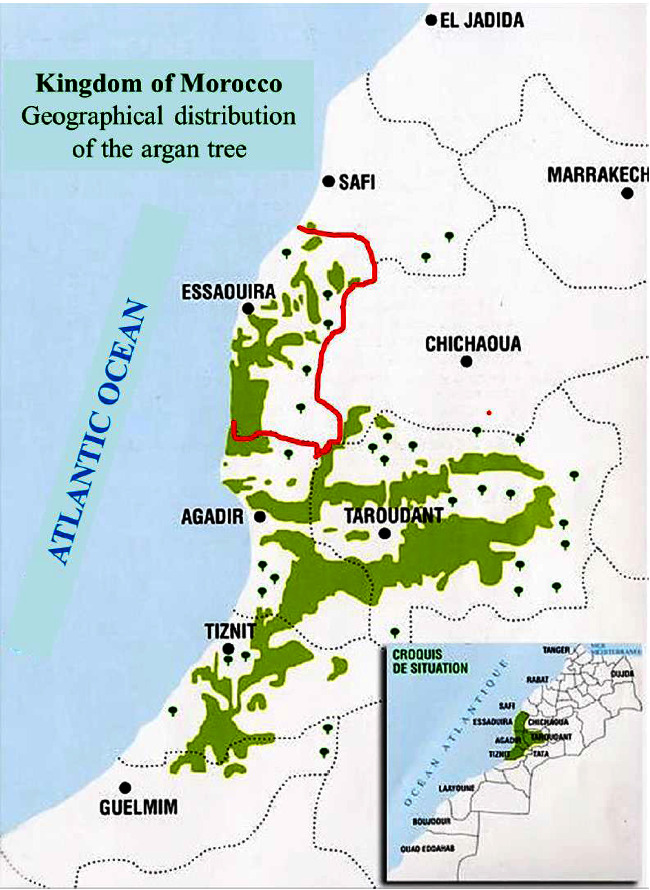
Map of argan forest distribution in southwestern Morocco [46].

This study was conducted in Essaouira province, located in the southwest of the Marrakech-Safi region, on the western slope of the High Atlas, along the Atlantic coast. The province borders Safi province to the North, Wilaya of Agadir and the province of Taroudant to the South, and Chichaoua province to the east (Figure 1). Essaouira province experiences a semiarid climate according to the Köppen–Geiger classification, with an annual rainfall of 342 mm, and average minimum and maximum temperatures of 11.0°C and 23.3°C, respectively. The province covers an extensive area of 649.115 hectares (6335 km^2^) with a population of 450.527 inhabitants. It is administratively subdivided into 57 communes, among which five are urban centers and the remaining 52 are rural. The population density averages around 71 inhabitants/km^2^, with 22.4% living in rural areas and 77.6% in urban zones [47]. The main vegetations consist of the argan tree (*Argania spinosa*), the barbary thuja (*Tetraclinis articulata*), and the red juniper (*Juniperus phoenicea*), either individually or in mixed formations.

#### 2.1.2. Data Collection

An ethnopharmacological survey was carried out in the province of Essaouira from February to April 2023, targeting individuals who are either native or have lived there for a long time immersing themselves in the local culture.

The methodology adopted for data collection was along the guidelines established by Martin [48], applying a stratified random sampling approach to ensure the representativeness of different demographic groups. A total of three hundred and twenty-five informants (*n* = 325), aged between 18 and 89 and from different socioeconomic backgrounds, were selected and interviewed, using a semistructured questionnaire. The interviews were without time constraints or pressure to encourage natural responses. Each interview lasted 40–60 minutes, gathering in-depth qualitative data on the cultural significance, traditional practices, and therapeutic applications of the argan tree.

The questionnaire was designed to collect a wide range of information, including demographic data on the informant (such as gender, age, family status, level of education, socioeconomic background, and source of information), as well as details concerning the uses of the argan tree for therapeutic, cosmetic, and culinary purposes. Indeed, specific aspects were gathered, namely, applications, applications, parts used, preparation methods, modes of administration, therapeutic indications, types of medicine, use in food, frequency, and reasons for use, as well as satisfaction levels and cultural traditions surrounding the use of the argan tree.

The questionnaire was prepared in Arabic (native language) and French (first foreign language), with explanations provided in Amazigh as needed. All participants received a clear explanation of the objectives of the survey before starting the interviews, thereby guaranteeing informed consent and ethical conduct.

### 2.2. Antioxidant Potential of Different Parts of Argan Trees

#### 2.2.1. Plant Material and Extraction Process

Various parts of the argan tree (*Argania spinosa* (L.) Skeels var. *apiculata*), including leaves, kernels, and nut shells, were collected in March 2023, from Essaouira region (31°26′49.0″ N 9°43′39.5″ W). Simultaneously, argan oil and press cake were obtained from an argan oil production cooperative in the same region, using fruit from the same trees.

The collected leaves, kernels, nut shells, and argan cake were dried at 40°C for three days and ground to powder (in an electric blender). Then, preweighed quantities of powder from each sample were washed three times with hexane to remove the lipid fraction.

Phenolic compounds were subsequently extracted from these preparations, along with a weighed argan oil, using a methanol/water mixture (80/20, v/v) three times. The mixtures were filtered, and the final extracts were evaporated and stored at 4°C until analysis [36, 49, 50].

#### 2.2.2. Total Phenolic Content Determination

Total phenolic content (TPC) was assessed colorimetrically using the Folin–Ciocalteu reagent following the method described by Slinkard and Singleton [51], with some modifications. Indeed, a 0.5 ml aliquot of the extracts was assayed with 2.5 ml of Folin–Ciocalteu reagent (10%, v/v) and 2 ml of sodium carbonate (20%, w/v). After incubation for 1 h at room temperature in the dark, the absorbance was read at 765 nm against a blank. Gallic acid was used as a standard for the construction of the calibration curve. The TPC values are expressed as milligrams of gallic acid equivalent per gram of plant materials of dry weight (mg GAE/g DW) for leaves, kernels, nut shells, and argan press cake samples, while for argan oil samples, they are given in milligrams of gallic acid, per kilogram of oil weight (mg GA/kg oil). All measurements were carried out in triplicate.

#### 2.2.3. DPPH Radical Scavenging Assay

The ability of the argan extracts for scavenging the stable 1,1-diphenyl-2-picrylhydrazyl (DPPH) free radical was assessed as previously described by Von Gadow et al. [52], with minor modifications. A 200 *μ*l of each methanolic solution of argan phenol extracts at different concentrations was added to 3 ml of methanolic solution of DPPH (0.004%). The mixture was shaken vigorously and incubated in the dark at room temperature for 60 min. The absorbance was then measured at 517 nm using a spectrophotometer against methanol as a blank. The scavenging activity, corresponding to the inhibition percentage of DPPH discoloration, was determined by the following formula:

(1) $$\begin{array}{c} \%\;i\text{nhibition}=\left(\frac{\left(A_{\text{control}}-A_{s\text{ample}}\right)}{A_{\text{control}}}\right)\times 100, \end{array}$$

where *A*_control_ is the control absorbance and *A*_sample_ represents the sample absorbance.

Gallic acid was used as standard controls for comparison. Measurements were repeated three times.

The IC_50_ value was calculated from the graph plotting inhibition percentage versus extract concentration and denotes the sample concentration required to scavenge 50% of DPPH radicals.

#### 2.2.4. Ferric Reducing Antioxidant Power (FRAP) Assay

The FRAP assay relies on the ability of the antioxidants in question (argan phenolic extracts) to transform the ferric iron (Fe^+3^) present in the (K_3_Fe[CN]_6_) complex into the ferrous form (Fe^+2^). The protocol described by Bounatirou et al. [53] (with minimal adjustments) was used to evaluate the reductive potential of argan phenolic extracts whereby various concentrations of extracts (2.5 ml) were combined (mixed) with 2.5 ml of phosphate buffer solution (0.2 M, pH 6,6) and 2.5 ml of 1% potassium ferricyanide (K_3_Fe[CN]_6_). The mixtures were incubated at 50°C for 20 min; then, after cooling at room temperature, 2.5 ml of 10% trichloroacetic acid was added to bring the reaction to a halt. After centrifuging the mixtures at 3000 rpm for 10 min, the upper layer fraction of each solution (2.5 ml) was then mixed with 2.5 ml of distilled water and 0.5 ml of ferric chloride (0.1% w/v). Absorbance was then measured using a spectrophotometer (700 nm) against a blank containing all reagents without phenolic extracts after being incubated under identical conditions. Higher absorbance indicates greater reducing power, and results were expressed as IC_50_ (mg/ml), which is the effective concentration corresponding to 0.5 absorbance. Ascorbic acid was used as a reference compound. Measurements were repeated three times.

### 2.3. Data Analysis

Ethnopharmacological data were analyzed to study the sociodemographic characteristics of the investigators, therapeutic uses, plant parts used, and modes of preparation and administration. For the antioxidant activity of argan phenolic extracts, all analytical determinations were carried out in triplicate, and data are expressed as means ± standard deviation. The results obtained were statistically analyzed by descriptive analysis and an analysis of variance (ANOVA), using the “STATGRAPHICS Centurion XVII package” software followed by the Tukey test with a *post hoc* multiple comparisons threshold at 5%. Graphs were designed using GraphPad Prism software.

## 3. Results and Discussion

### 3.1. Ethnopharmacological Survey

#### 3.1.1. Sociodemographic Data

Analysis of the data collected from an ethnopharmacological survey of 325 respondents living in the province of Essaouira (Morocco) (Table 1) showed that 57.8% of argan users were women, compared with 42.1% of men. Several studies have evidenced similar trends, reporting that women have a deeper knowledge and more frequent use of medicinal species, including the argan tree, compared to men [27, 54, 55]. Katiri et al. [56] noted that, unlike men, women acquire this knowledge through regular observations from their mothers and grandmothers. This could be attributed to the increased interest shown among women in maternal health and their strong preference for using, in particular, argan oil for cosmetic purposes [22].

The use of argan and its products concerns all age groups. Indeed, 39.08% of respondents are aged between 20 and 30, followed by the {31–40} and <20 age categories (18.8% and 16.3%, respectively). The percentages of respondents in the {41–50}, {51–60}, and >60 groups are not negligible but do not exceed 9%. Our findings contradict previous surveys [24, 27, 57, 58], which reported that ethnomedical knowledge is mostly held by the elderly and that young people exhibit a decrease in their confidence in local products and especially in traditional medicine, whereas, in our case, we noted changes in the attitude of the local population toward local argan products. We noticed a rising interest among young people in using indigenous products for therapeutic and cosmetic purposes. This is not restricted to specific social groups and can be attributed to both personal experience and scientific knowledge.

People with a high tendency to use argan are predominantly married (51.4%) and single (39.1%), while divorced and widowed individuals are less represented (<5%). This corroborates the observations of Barkaoui et al. [20] and Bachar et al. [57] who suggested that married people tended to use the argan tree and medicinal plants in general for their care to minimize the onerous costs required by modern medicine.

Our sample of interviewees was also distinguished by a high proportion of educated people, with 49.54% being university graduates and 22.5% having received a secondary education. Persons with a primary education accounted for 14.1%, while illiterates represented only 13.8%. This contrasts with other studies that indicated that illiterate individuals use the argan tree [20, 27, 57] as well as other species [24, 58] more frequently compared to other categories.

This survey demonstrated that the informants were predominantly of intermediate socioeconomic level (87.1%), while the remainder was equally divided between low- and high-socioeconomic status. Concerning location, our findings displayed that 69.8% of informants lived in urban zones, while 30.1% were from rural areas, contradicting the statements of Barkaoui et al. [20], which claim that the use of medicinal plants decreases with urbanization.

The information received on the use of argan and its products for various purposes comes mainly from own experiences (57.2%), “literature and research” is ranked as the second source of information with 20.3%, followed by “doctors and pharmacists” (7.7%) and “herbalists” (4.6%). The increasing use of argan tree products among young and well-educated people may explain their preference for consulting scientific information, doctors, and pharmacists over herbalists.

#### 3.1.2. Uses, Used Argan Parts, Their Preparation, and Administration

**Figure 2:**
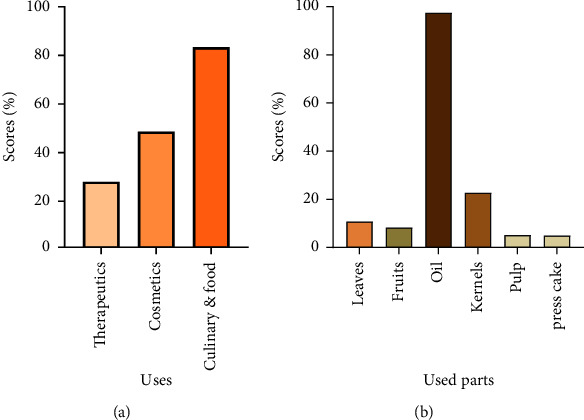
Breakdown of uses (a) and used parts (b) of the argan tree by the interviewed population in the province of Essaouira.

In the present investigation, we aimed to emphasize the various applications of the argan tree and its products, those parts most used by the local population, as well as the methods of their preparation and administration. The classification by category of use (Figure 2(a)) indicated that 83.4% of participants used argan or one of its products as a food or food ingredient. Cosmetic and therapeutic uses were ranked in 2^nd^ and 3^rd^ place by 48.6% and 28%, respectively. The data revealed that argan oil topped (97.5%) among the parts or products that respondents claimed to use. Interviewees declared using the other parts as well, but to varying extents: kernels (22.8%), leaves (10.8%), fruit (8.31%), pulp (5.2%), and press cake (4.9%) (Figure 2(b)). Similarly, Barkaoui et al. [27] reported in an ethnobotanical survey conducted on argan in Morocco's western anti-Atlas that oil extracted from argan almonds is the most widely used, albeit with a relatively lower amplitude than our result (72%). Nevertheless, the leaves have been documented as the commonly utilized component in many national and international ethnobotanical studies on medicinal and therapeutic plants [24, 57, 59–68].

**Figure 3:**
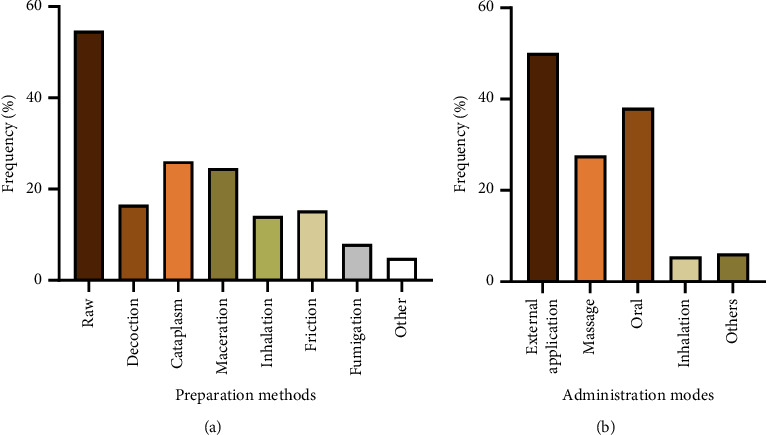
Frequency of preparation (a) and administration (b) modes for used parts of the argan tree by the interviewed population in the province of Essaouira.

In terms of preparation and depending on the intended use, several methods are applied to make administration easier.  Figure 3(a) illustrates that 54.8% of the local population consumes argan products in their raw state, aligning with our previous observation that argan oil is the preferred product. Cataplasm (26.1%) and maceration (24.6%) also appear to be favored for preparing argan materials. Then, in varying measures, other methods, namely, decoction, friction, and inhalation, were mentioned. All of these methods have been extensively recorded as being applied to different extents to extract the most active principles and reduce or eliminate their harmful effects, taking into account the specific traits of each plant and the indigenous knowledge available in each area [27, 60, 68–71].

Concerning administration mode, we observed that the local population uses external application (50.1%) as the prevailing choice, followed by the oral way (38.1%) and massage (27.7%). The other modes are represented by minor magnitudes (Figures 3(b)). Administration methods for argan products and by-products are mainly influenced by those most commonly used, their preparation methods, and envisaged applications [68].

#### 3.1.3. Therapeutic, Cosmetic, and Food Uses of Argan

**Figure 4:**
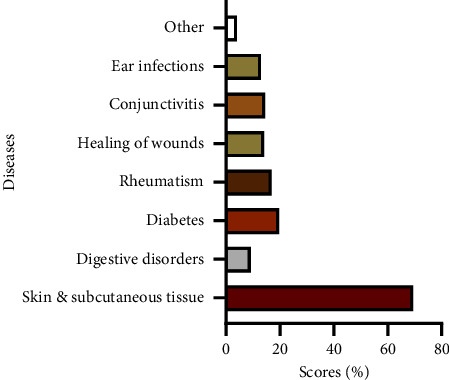
Distribution of diseases treated with parts of the argan tree according to the interviewed population in the province of Essaouira.

The present survey revealed that argan and its derivatives are used for different purposes and against a variety of diseases. The usage breakdown (Figure 4) illustrates that the treatment of skin and subcutaneous tissue issues (69.5%) dominates the use of argan in the province of Essaouira. Diabetes treatment and prevention comes second at 19.7%, and 15% of interviewees stated having used argan to cure ear infections, conjunctivitis, wounds, and rheumatism. Its use for digestive disorders accounted for 9.2%. Using argan products to treat skin and subcutaneous tissue problems has been extensively cited in many previous studies. Argan oil has been reported to be mainly used, alone or combined with other argan ingredients, to hydrate and care for both skin and hair [27], as well as to treat various skin conditions, including burns [72], scars [73], teenage acne [74], and chickenpox pustules [75]. Mechqoq et al. [10] mentioned the use of a mixture containing argan oil for treating mange. Argan seeds are mainly applied to treat eczema [24], sprains, wounds [18], skin burns [22], and hair care [18] and are also useful against internal diseases like gastritis, lung infections, cardiovascular disorders, and diabetes [10, 20]. Argan leaves have shown great potential against gastritis and ulcers [22], diabetes [56], gastritis, dysentery, headaches, and fever [24]. Alaoui [73] reported the application of argan pulp to treat urticaria, mycosis, parasites, and scabies. Argan cake served to remedy dermatitis, sprains, and skin lesions [18].

**Figure 5:**
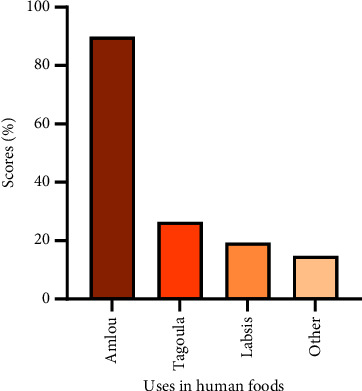
Breakdown of argan's uses in human foods according to the interviewed population in the province of Essaouira.

For its use in human food, argan oil is the only derivative that has long been consumed directly as a food in its raw state, as well as an ingredient for preparing authentic foods. The most widely used food (89.8%) is Amlou, which is a mixture of argan oil, almonds, and honey. Other foods based on argan oil are prepared and served by the local population, notably, Tagoulla (26.5%) and Labsis (19.4%) (Figure 5). “Tagoulla” is a traditional preparation based on barley or corn semolina, typically seasoned with argan oil. “Labsis” is generally composed of toasted barley flour, honey, hard-boiled eggs, salt, and argan oil [27]. Moukal et al. [18] stated that argan oil is a key ingredient in “tagines,” “couscous,” “harira,” soups, and salads or in more original recipes, such as “Tagoulla,” “Lamris,” “Toumit,” “Labsis,” or “Amlou.”

**Figure 6:**
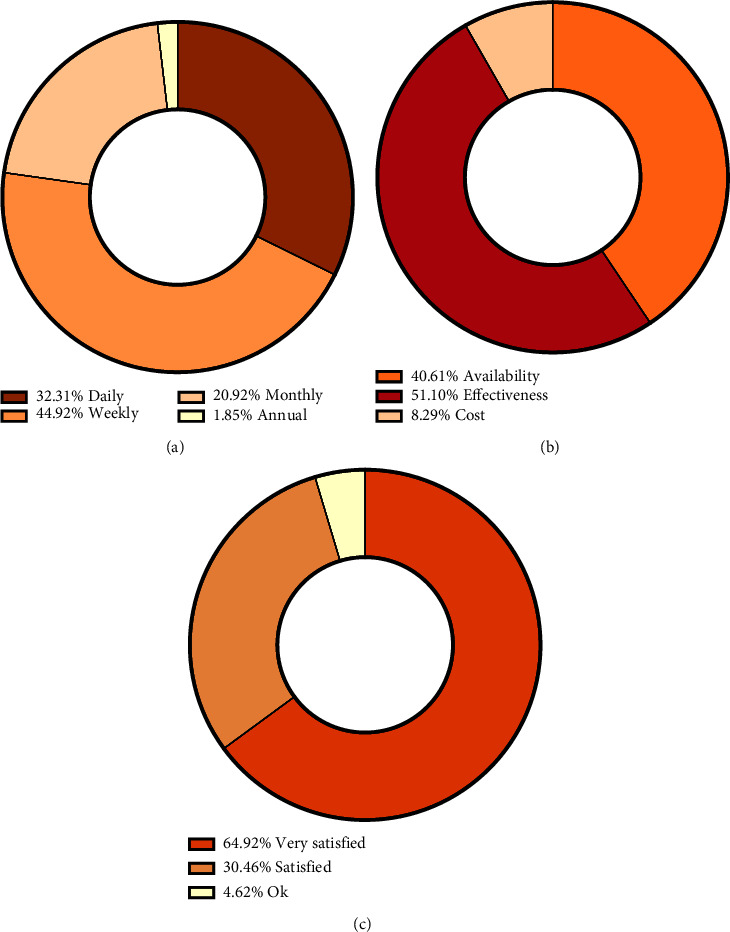
Frequency (a), reasons for use (b), and level of satisfaction (c) of informants using argan products (in Essaouira province).

The local population was also surveyed regarding their reasons for using argan and its products (Figure 6). The responses were primarily divided between the effectiveness (56.9%) of argan and its products in therapeutic, cosmetic, and dietary applications, as well as its availability (45.2%) in the studied region. The effectiveness of argan-derived products can be attributed to their high content of antioxidants, vitamins, and essential fatty acids [10, 76, 77]. The argan tree is an indigenous and typical species of the southwestern Moroccan landscape, including the province of Essaouira. This natural abundance may explain the availability of argan products, allowing consumers to easily access them and incorporate them into their daily routines.

This survey has affirmed the importance of *Argania spinosa* L. Skeels as a treasured asset in the cultural heritage and traditional practices of the local community. Indeed, it has enabled a better understanding of the significance of the medicinal, therapeutic, and dietary uses of this plant, while evidencing an increased adoption of these practices among young and well-educated people.

### 3.2. Antioxidant Potential of Different Parts of Argan Trees

**Figure 7:**
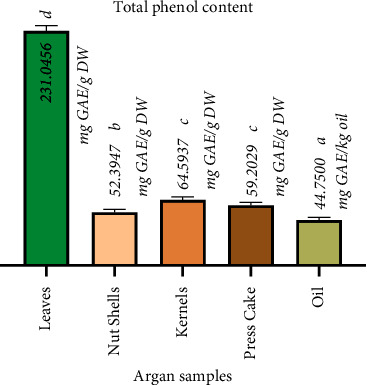
Total phenol contents in extracts from various parts of *Argania spinosa* (L.) Skeels collected in Essaouira province. The values are the mean of three determinations ± standard deviation. Means followed by the same letter are not significantly different (Tukey's test, *p* < 0.05).

Various samples of argan (*Argania spinosa* L. Skeels) including leaves, kernels, nut shells, argan oil, and press cake were collected, prepared, and analyzed for their total phenolic content (TPC). The average values for TPC for the different samples are presented in Figure 7. Our findings affirm that argan material is a promising source of phenolic compounds, although there are significant variations among the different samples analyzed. Argan leaves showed a substantially higher TPC (>4 times; 231.046 ± 5.090 mg GAE/g DW) compared to kernels (64.594 ± 2.789 mg GAE/g DW), nut shells (52.395 mg ± 1.727 GAE/g DW), and press cake (59.203 ± 2.762 mg GAE/g DW). Argan oil, on the other hand, exhibited an average TPC of 44.570 ± 1.548 mg GAE/kg oil. The TPC measured in the present work falls within the range recorded in many similar investigations. It should be noted that most of the studies conducted on the argan tree have mainly focused on the leaves, rather than on other parts and products. In line with our data, TPC in argan leaves has been reported to range from 210 to 378 mg GAE/g DM [29, 78, 79]. However, it is important to note that other reports have indicated values significantly higher [36, 44] or much lower [80, 81] than those observed in our study. For argan fruits, TPC has also revealed varied results. For instance, El Idrissi et al. [44] found a relatively high TPC of 207.5 mg GAE/g DW in kernels collected from the Essaouira region (which is the same study area as ours), while another study [38] conducted on kernels from Chtouka Ait Baha (Morocco) showed a much lower TPC level (8.2 mg AGE/g DW). Values for argan oil also widely differed among studies, ranging from 13.2 mg AG/kg to 112.67 mg GA/kg, as reported by Rojas et al. [17] and Kamal et al. [82], respectively. Our results regarding TPC in kernels and argan oil match those observed in Atifi et al. [49] and Dakiche et al. [29], respectively. In terms of argan press cake, the TPC in our samples was largely higher than those observed by El Monfalouti et al. [38] but lower than the scores of Rojas et al. [17]. The TPC discrepancy among fruit constituents, press cake, and argan oil can be attributed to the extraction process, which involves crushing, malaxation, and separation. During this process, only some of the phenols present in the fruit are transferred to its oil. The higher TPC values observed in leaves, compared to the other argan samples, can be attributed to their specific functions. In leaves, phenolic compounds primarily act as a protection and defense mechanism against environmental influences, including herbivores, insects, pathogens, UV rays, and environmental stressors [49, 83]. However, it is worth stating that the TPC variability recorded in various studies can be assigned to several factors, notably genotype, geographical origin, agricultural practices, age of the tree, harvesting time, and climatic conditions [36, 83–89].

**Figure 8:**
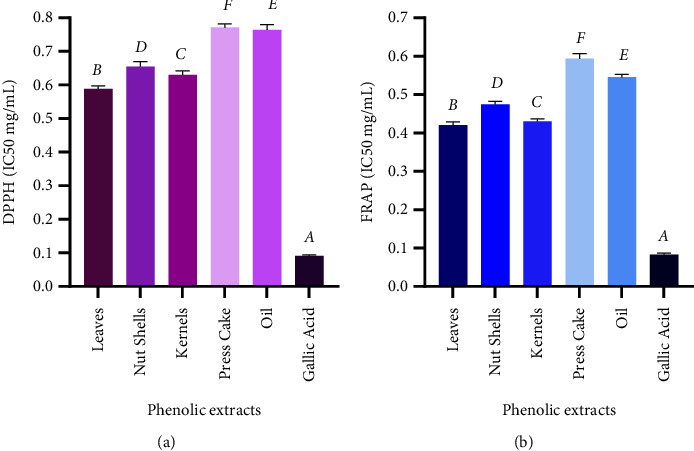
Antioxidant activities according to DPPH (a) and FRAP (b) tests of different extracts from different parts of *Argania spinosa* (L.) Skeels collected in Essaouira province. The values are the mean of three determinations ± standard deviation. Means followed by the same letter are not significantly different (Tukey's test, *p* < 0.05).

In this investigation, we carried out two different tests to assess the antioxidant capacity of phenolic extracts derived from various parts of the argan tree, namely, leaves, nutshells, almonds, press cake, and oil. These tests are designed to assess different antioxidant processes. The first was the DPPH assay, which provides information about the ability of antioxidants to neutralize free radicals by either donating electrons or hydrogen atoms. The second was the FRAP test, based on the power of antioxidants to reduce the ferric iron (Fe^+3^) contained in the [K_3_Fe(CN)_6_] complex to the ferrous form (Fe^+2^). The results from both assays were expressed as IC_50_ (mg/ml), indicating the concentration of antioxidants required to inhibit or reduce the specified activity by 50%, and are summarized in Figure 8.

Our findings revealed that argan material and products can be an excellent and promising source of natural antioxidants. Indeed, all of the extracts, as well as the gallic acid standard, exhibited substantial free radical scavenging and reducing power, with a dose-related pattern. These observations are in harmony with those documented in earlier studies [21, 29, 37, 49, 78, 80, 90–92], reinforcing that argan-derived compounds have notable antioxidant properties. In addition, statistically significant differences (*p* < 0.001) were recorded among the IC_50_ mean values of the various argan extracts for both assays. Specifically, the leaf phenolic extract displayed the greatest free radical scavenging potential (IC_50_ = 0.589 ± 0.005 mg/ml) and the most effective reducing power (IC_50_ = 0.420 ± 0.005 mg/ml). In contrast, the data obtained indicated that the argan oil samples had the lowest antioxidant capacities, with IC_50_ values of 0.772 ± 0.009 mg/ml for the DPPH assay and 0.594 ± 0.009 mg/ml for the FRAP test. The extracts obtained from argan kernels, nut shells, and press cake also demonstrated notable free radical neutralizing capacities, with mean IC_50_ values of 0.631 ± 0.007, 0.655 ± 0.008, and 0.764 ± 0.008 mg/ml, respectively. Similarly, these extracts displayed effective reducing capacities, as indicated by their IC_50_ values of 0.430 ± 0.004 mg/ml, 0.474 ± 0.005 mg/ml, and 0.546 ± 0.008 mg/ml, respectively. Although low concentrations of argan phenolic extracts resulted in significant free radical scavenging and reducing activities, those recorded for the gallic acid standard were even stronger (IC50 values were 0.091 ± 0.002 and 0.083 ± 0.002 mg/mL for both DPPH and FRAP tests, respectively). In agreement with our results, El Idrissi et al. [44] reported that leaves had a higher antioxidant capacity than other argan plant parts (seed, pulp, kernel, and branch) in an investigation conducted on samples collected in the Essaouira region. Similarly, another study carried out in Algeria indicated that leaf extracts offered higher antioxidant potential than argan pulp and oil [29]. El Monfalouti et al. [38] noted that the radical scavenging activity of polyphenols extracted from press cake appeared to be less effective than that of shells and kernels of the argan fruits from Ait Baha (Chtouka-Ait Baha, Morocco). This difference in antioxidant potential among different parts of the argan tree is likely attributed to their distinct chemical compositions. Argan leaves may contain higher levels of specific phytochemicals with notable biological properties compared to kernels, press cake, and argan oil [74]. Extraction methods involving exposure to heat, light, and oxygen can potentially degrade antioxidants, especially in argan oil and press cake [93]. Stagos et al. [94] suggested that the antioxidant activity depends on the quality, rather than the quantity of phenolic compounds, and may also be attributable to other phytochemicals besides polyphenols. The same authors suggested a synergistic effect between phenolic compounds and other phytochemicals. Furthermore, the antioxidant properties of phenolic compounds are a function not only of their quantity but also of their structure and nature [95–97].

## 4. Conclusions

The ethnopharmacological survey conducted in the province of Essaouira revealed the central role of argan products, particularly argan oil, in the traditional medicine, cosmetics, and cuisine of the local population, considering them a fundamental cultural element. Moreover, findings arising from the present study revealed that argan products exhibited promising levels of total phenolic content and antioxidant capacities, evidenced by DPPH and FRAP assays. These results lend support to the population's uses of argan products to treat a variety of therapeutic indications, including skin and subcutaneous tissue, digestive disorders, diabetes, rheumatism, wound healing, conjunctivitis, and ear infections. The data gathered from the present investigation provides a strong basis for further research into the therapeutic and cosmetic potentials of argan-derived products. The antioxidant properties of these products suggest their possible use as safer natural alternatives to synthetic antioxidants, especially in human foods, whether for enhancing nutritional quality or extending shelf life. Additionally, more extensive exploration of the phytochemical composition and biological activities of the argan tree could lead to improved sustainable agricultural practices and the discovery of new applications. Overall, these findings open up promising avenues for harnessing this traditional natural resource to meet a growing array of needs. Despite their encouraging antioxidant activity, further research is needed to validate argan extracts due to the inconsistency of extraction methods and the limitations of *in vitro* assays. Clinical trials are therefore essential to assess the bioavailability, safety, and efficacy of these extracts.

## Figures and Tables

**Table 1:** Profile of argan users in Essaouira province (gender, age, family situation, education level, socioeconomic situation, location, and source of information).

Sociodemographic variables		Number (and %) of user informants	
Gender	Female	188	57.8
	Male	137	42.1

Age category	<20	53	16.3
	20–30	127	39.1
	31–40	61	18.8
	41–50	26	8.0
	51–60	29	8.9
	>60	27	8.3

Family situation	Married	127	39.1
	Single	167	51.4
	Divorced	15	4.6
	Widower	16	4.9

Education level	University	161	49.5
	Secondary	73	22.5
	Primary	46	14.1
	Illiterate	45	13.8

Socioeconomic situation	High	20	6.1
	Intermediate	283	87.1
	Low	22	6.8

Location	Urban	227	69.8
	Rural	98	30.1

Information source	Literature and research	66	20.3
	Pharmacists and doctors	25	7.7
	Own experiences	186	57.2
	Herbalists	15	4.6
	Others	33	10.1

## Data Availability

The datasets used and analysed during the current study are available from the corresponding author on reasonable request.
